# Left Ventricular Pressure Estimation Using Machine Learning-Based Heart Sound Classification

**DOI:** 10.3389/fcvm.2022.763048

**Published:** 2022-05-25

**Authors:** Philip Westphal, Hongxing Luo, Mehrdad Shahmohammadi, Luuk I. B. Heckman, Marion Kuiper, Frits W. Prinzen, Tammo Delhaas, Richard N. Cornelussen

**Affiliations:** ^1^Department of Physiology, Cardiovascular Research Institute Maastricht (CARIM), Maastricht, Netherlands; ^2^Bakken Research Center, Medtronic, plc, Maastricht, Netherlands; ^3^Department of Biomedical Engineering, Cardiovascular Research Institute Maastricht (CARIM), Maastricht, Netherlands

**Keywords:** heart sound, hemodynamics, cardiac resynchronization therapy, artificial intelligence, machine learning, animal, epicardial acceleration

## Abstract

**Objective:**

A method to estimate absolute left ventricular (LV) pressure and its maximum rate of rise (LV dP/dtmax) from epicardial accelerometer data and machine learning is proposed.

**Methods:**

Five acute experiments were performed on pigs. Custom-made accelerometers were sutured epicardially onto the right ventricle, LV, and right atrium. Different pacing configurations and contractility modulations, using isoflurane and dobutamine infusions, were performed to create a wide variety of hemodynamic conditions. Automated beat-by-beat analysis was performed on the acceleration signals to evaluate amplitude, time, and energy-based features. For each sensing location, bootstrap aggregated classification tree ensembles were trained to estimate absolute maximum LV pressure (LVPmax) and LV dP/dtmax using amplitude, time, and energy-based features. After extraction of acceleration and pressure-based features, location specific, bootstrap aggregated classification ensembles were trained to estimate absolute values of LVPmax and its maximum rate of rise (LV dP/dtmax) from acceleration data.

**Results:**

With a dataset of over 6,000 beats, the algorithm narrowed the selection of 17 predefined features to the most suitable 3 for each sensor location. Validation tests showed the minimal estimation accuracies to be 93% and 86% for LVPmax at estimation intervals of 20 and 10 mmHg, respectively. Models estimating LV dP/dtmax achieved an accuracy of minimal 93 and 87% at estimation intervals of 100 and 200 mmHg/s, respectively. Accuracies were similar for all sensor locations used.

**Conclusion:**

Under pre-clinical conditions, the developed estimation method, employing epicardial accelerometers in conjunction with machine learning, can reliably estimate absolute LV pressure and its first derivative.

## Introduction

Heart failure is a major public health concern for healthcare systems that struggle to treat the ~37 million patients worldwide (1). The Western world alone experiences more than 1 million hospitalizations each year, a number that rapidly increases due to the aging population groups (2–4). Cardiac decompensation results in more frequent/prolonged hospitalization of patients, causing treatment costs to increase while reducing the quality of life and life expectancy (5, 6). Therefore, tools that may prevent hospitalizations in patients with heart failure would be beneficial (7).

Continuous hemodynamic monitoring improves the conventionally static behavior of current treatment methods, reducing the need for follow-up visits or hospitalization. Several implantable devices have been developed to optimize therapy and identify decompensation episodes in an early stage (7, 8).

For this purpose, the measurement of left ventricular pressure (LVP) or its first derivative would be the first choice. However, the required invasive intervention and potential complications like drift, sensor overgrowth, leakage, and embolization make this approach less suitable for chronic applications (9). As a surrogate for LV function, the Chronicle^TM^ system measures RV pressure. Clinical studies show promise in reducing readmission rates due to congestive heart failure (10). Similarly, the CardioMEMS™ system consists of a small pressure-sensing device that is implanted directly into the pulmonary artery (11). However, these systems are expensive, stand-alone devices and it is unclear how right-sided measurements are related to LV function (10).

An alternative method is the use of accelerometers. These are small mechano-sensors that can easily be integrated with, for example, devices and catheters. The best-known example is currently the Peak Endocardial Acceleration (PEA, later renamed as SonR) system that uses an accelerometer integrated into an implantable right atrial or RV pacing lead (12) to measure the amplitude of the first heart sound (13, 14). The RESPOND-CRT trial demonstrated that this automatic, SonR sensor-guided optimization of pacemaker therapy was safe and slightly superior to the conventional Echo-guided optimization (15). However, the SonR system does not provide absolute pressures.

In recent years machine learning (ML) has rapidly developed. ML is a computational discipline focused on building algorithms that model or recognize (complex) patterns or characteristics within large amounts of data. It is used increasingly in the heart failure space either prognostically or as in this article diagnostically (16). A previous study has indicated successful classification of heart sounds for valvular diseases *via* machine learning-based methods (17). We hypothesized that machine learning may improve the analysis of accelerometer data to the extent that also absolute values of hemodynamic parameters can be estimated.

For this purpose, animal experiments were performed, where gold standard pressure and accelerometer measurements were recorded under widely varying hemodynamic conditions and at different cardiac anatomic positions. Automatic accelerometer classification was facilitated *via* beat-by-beat segmentation of accelerometer and pressure signals. After extraction of acceleration and pressure-based features, a model was trained using machine learning to estimate absolute values of LVPmax and its maximum rate of rise (LV dP/dtmax).

## Methodology

### Study Overview

A total of five acute open chest (weighing 60–65 kg) experiments in pigs were performed in accordance with Dutch Law on Animal Experimentation and the European Directive for the Protection of Vertebrate Animals used for Experimental and Other Scientific Purposes. The protocol was approved by the Central Committee for Animal experiments (CCD) in the Netherlands and the Animal Experimental Committee of Maastricht University.

### Experimental Setup

The animals were premedicated with Zoletil (5–8 mg/kg) whereafter anesthesia was induced using thiopenthal (5–15 mg/kg IV). Propofol (2.5–10 mg/kg/h), sufentanyl (4-8 μg/kg/h), and rocuronium (0.1 mg/kg/h) were given at regular intervals to maintain the anesthesia. Heparin was given throughout the experiment as an anticoagulant to suppress blood clotting.

**Figure 1:**
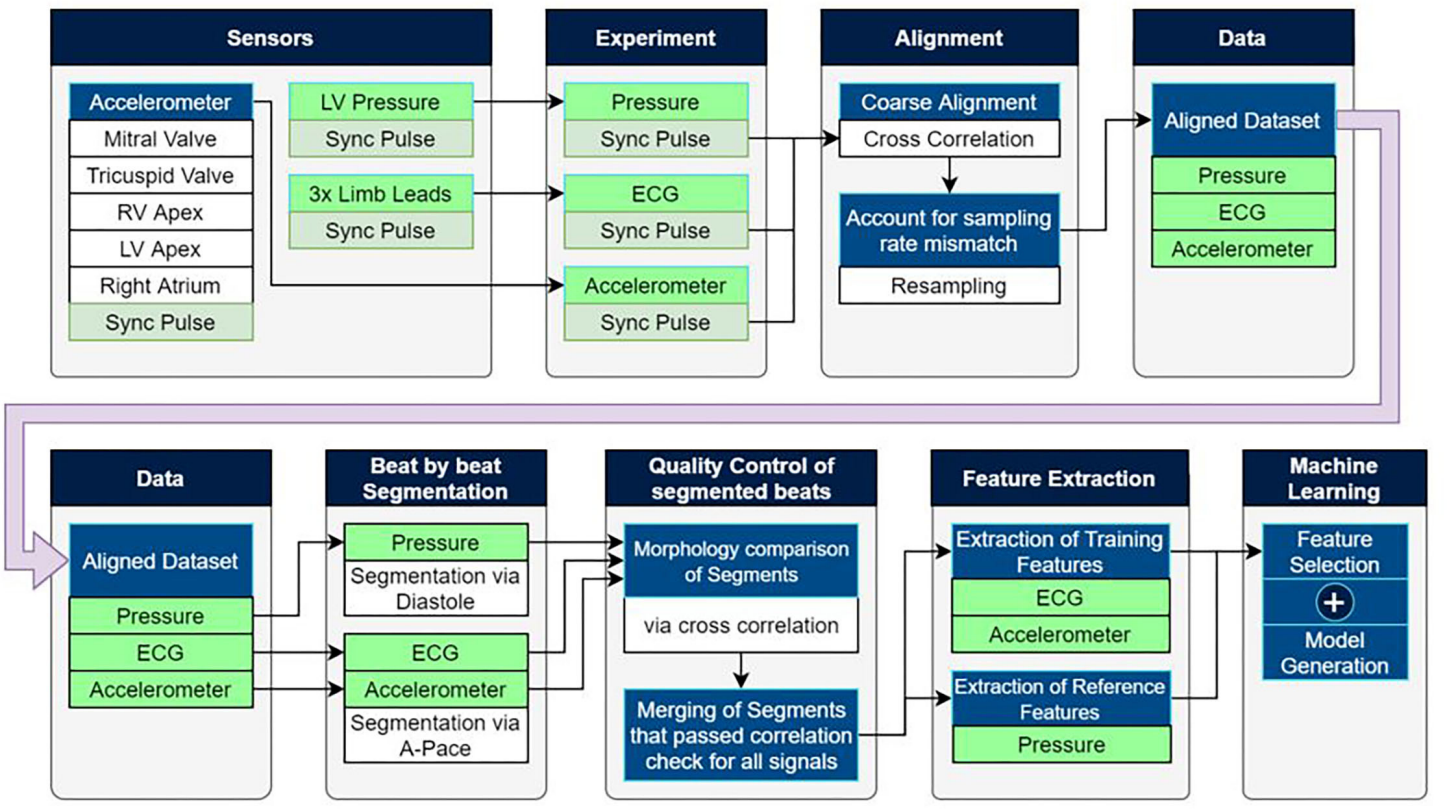
Experimental setup as well as the post-processing pipeline used to extract training data for machine learning based estimation models.

Data acquired during the experiments consisted of electrocardiogram (ECG), LVP, and epicardially measured acceleration signal. An overview of the experiment and the data analysis are depicted in Figure 1.

ECG measurements were acquired using the limb-leads. Pressures were measured using 7F catheter-tip manometers (CD-Leycom, Zoetermeer, the Netherlands). Under fluoroscopic guidance, pressure catheters were inserted *via* the carotid artery and the jugular vein into both the right and left ventricles as well as the right atrium. In addition, a pressure catheter was placed in the aorta.

Pacing leads were inserted transvenously into the right atrium and right ventricle (RV), while an LV lead was placed epicardially on the LV free wall, using a plunge electrode introduced into the thorax through a small incision. Pacing thresholds were evaluated on an individual basis for each electrode.

Radiofrequency ablation of the atrioventricular node was used to an create atrio-ventricular block (AVB). The process made use of an ablation catheter (MarinR, Medtronic plc.) and a radio frequency power generator (Atakr, Medtronic plc.)was performed under fluoroscopic guidance. After the creation of AVB, ventricular pacing was initiated for hemodynamic stability.

Mechanical data was acquired *via* custom-made epicardial mechano-sensors, designed to facilitate recordings of high acquisition resolution and sampling rate while keeping the overall size of the sensor to a minimum dimension of 3.3 mm (X) ^*^ 5 mm (Y) ^*^ 1.6 mm (Z). Each sensor package consisted of 3^*^ Hall-effect-based accelerometers, perpendicularly aligned to each other, sensing at a resolution of 16-bit at a sampling rate of 1,000 Hz. Each sensor was paired with a single analog to digital converter (ADC) (a total of 3), allowing synchronous capture of data. The data were recorded in a range of ±4 g to accommodate a minimum range of ±3 g when accounting for gravity with a 16-bit digitization resolution. A custom device was made to facilitate the simultaneous acquisition of the mechano-sensors and to allow simple integration into existing systems *via* a shared synchronization pulse.

### Alignment

Alignment of the datasets recorded from the ECG, pressure and mechano-sensors was performed *via* a shared synchronization pulse that is broadcasted by the acquisition device. Each system/device connected to the synchronization pulse generated a tracing on a separate channel and was contained within each of the datasets at the end of the experiment. Using this signal, dropped/duplicate samples or mixed sampling frequency systems can be recognized, and the section of the signal was resampled automatically.

Embedded in a silicone suturing fixture, the mechano-sensors were sutured onto the epicardium through a small thoracic incision. The dimensions of the fixture allowed only for one-sided mounting of the sensor and enforced correct/consistent orientation to maintain signal uniformity between the experiments by aligning the sensor's *Z*-axis perpendicular with the tissue surface. The thorax was partially closed to minimize the effect on the animal's hemodynamics after the placement of the sensors. A small hole was retained for the cabling without hermetic sealing and therefore the experiments remained “open chest.”

A total of five sensors were attached to the tissue at the locations shown in Figure 1, being the LV and RV apex, RV and LV free wall close to the mitral and tricuspid valves, as well as the right atrium.

### Experimental Protocol

Pacing protocols consisted of RV and biventricular (BiV) pacing with incrementally increasing atrioventricular (AV) pacing delays, ranging from 50 to 300 ms, and incrementally increasing interventricular (VV) delays ranging from −150 to +150 ms. The pacing protocol was performed in DDD mode and repeated under different hemodynamic loading conditions. Dobutamine (DOB) was used to increase cardiac function and its dose was adjusted to reach approximately twice the baseline LV dP/dt max value. After a sufficient weaning period from the dobutamine, the animals were ventilated with the cardiovascular depressant isoflurane (ISO) to decrease the baseline LV dP/dt(max) value to around half of the baseline value. Pacing protocols were repeated during both dobutamine and isoflurane administration. Each setting maintained a 60 s recording time unless the applied pacing setting appeared detrimental to the animal's hemodynamic state.

### Data Analysis

Please note that more details regarding the data analysis can be found in the Supplementary Material. The experimental setup made use of multiple standalone recording devices with different sampling rates which shared an auxiliary synchronization pulse as shown in Figure 1. Matching the pulse between devices allowed for precise temporal alignment of the ECG, pressure, and mechanosensory data.

The automated post-processing pipeline segmented the input signals into individual cardiac cycles. Training features were extracted from the ECG and acceleration signals, while reference features were extracted from the LVP signals. A morphological cross-correlation analysis was performed within each pacing setting to identify the largest coherent group of cardiac cycles and remove deviating beats containing artifacts or non-typical paced beats.

ECG analysis employed a band-pass filter to remove the DC-offset and the effects of respiration drift. Individual cardiac cycles were segmented by identification of atrial pacing spikes after which training features were extracted from the signal.

As acceleration signal's energy, during occurrences of the heart sounds, of S1 and S2 was found to be negligible for frequencies above 250 Hz, a (10–250 Hz) bandpass filter was used to remove DC-offset and high-frequency noise.

**Figure 2:**
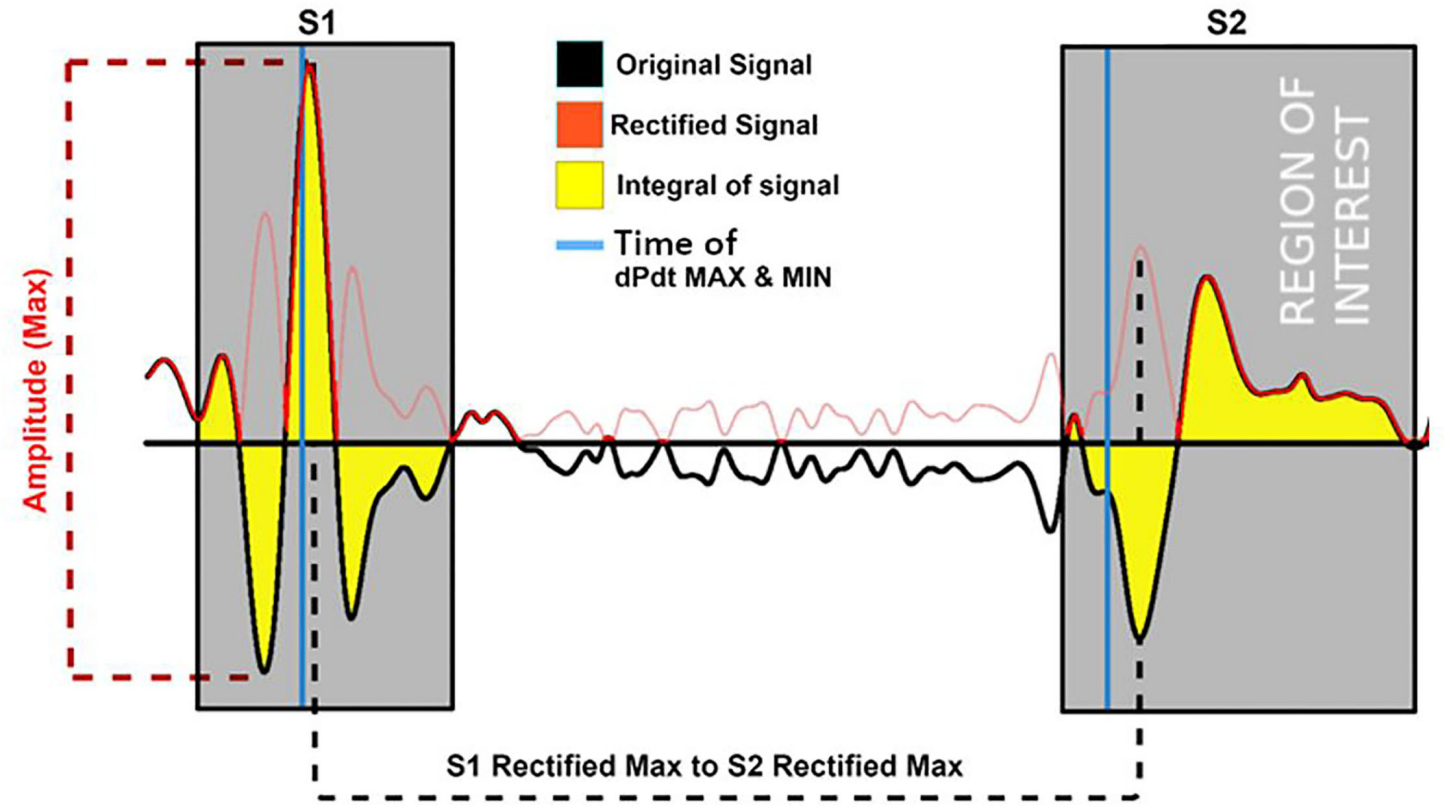
Examples of the training features extracted from the accelerations signal (black) and its rectified version (red). Features depicted are the maximum amplitude of the signal; its integral and the location time duration between the rectified S1 and S2 maximum of the rectified (negative and positive as positive) signals. Gray area = region of feature extraction.

**Figure 3:**
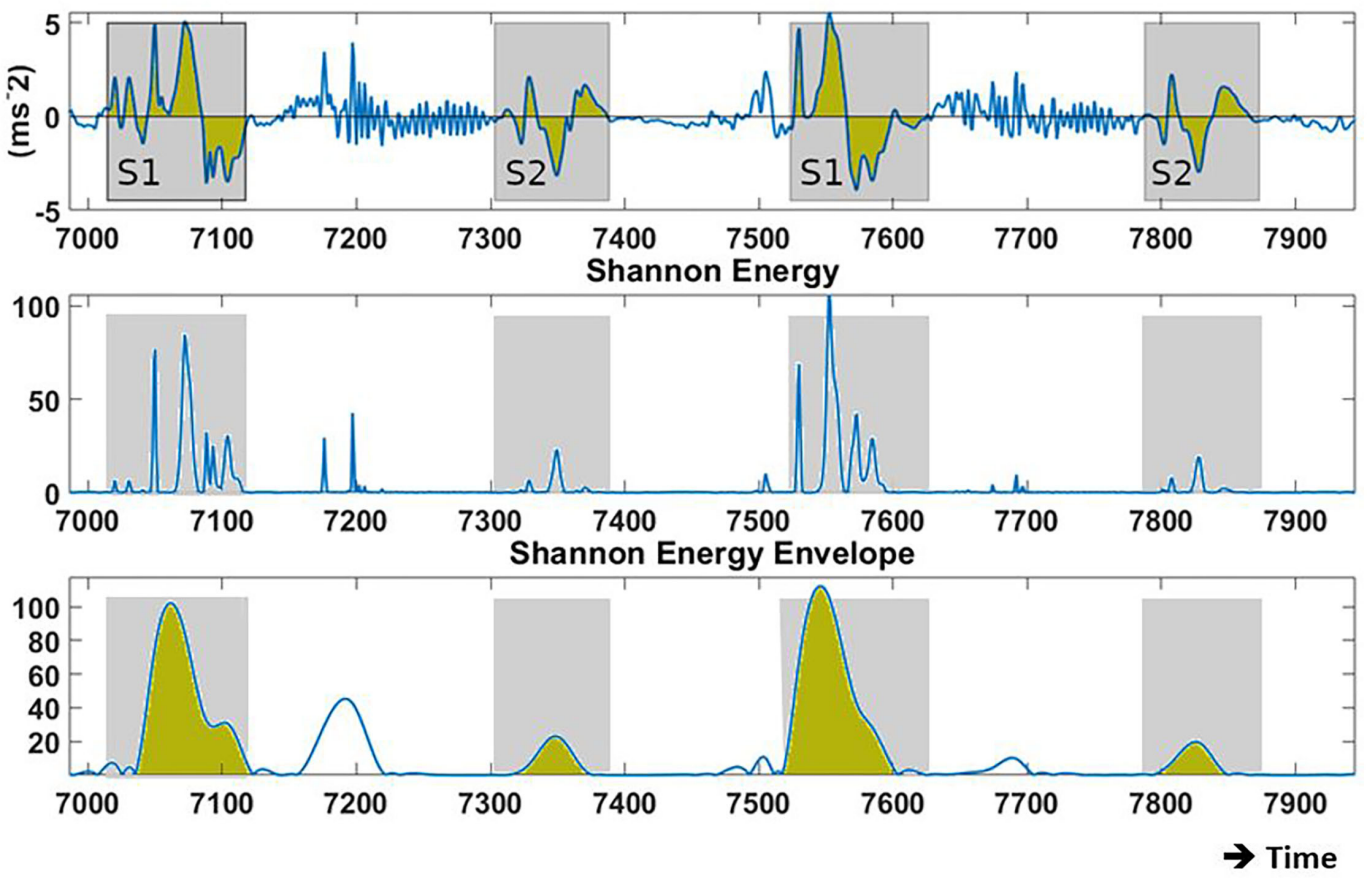
Examples of the recorded baseline acceleration signal (Bi-Ventricular (BIV) pacing, Atrio-Ventricular (AVd): 150 ms) and its Shannon energy from which training features are derived. (Top) acceleration during two cardiac cycles in proximity to the mitral valve. (Center) Shannon energy of the original signal, pronouncing low intensity amplitudes over high intensity amplitudes. (Bottom) Displays the envelope based on the Shannon energy. Blue line = raw signal; Yellow area = signal integral; Gray area = region for feature extraction.

The atrial pacing spike was used to segment each beat of the acceleration signal post-alignment. Features reflecting amplitude, timing, and energy of S1 and S2 were extracted from the signals for the machine learning process. A selection of the 17 (based upon literature) predefined training features extracted from the acceleration signal are shown in Table 1. Figures 2, 3 depict examples of the features shown in Table 1.

**Table 1:** Training features that are extracted from regions of interest (Figure 3) located around S1 & S2 of the acceleration and ECG signals.

	**Features**	**Code**	**Details**
Amplitude	Amplitude (max)	A1 or A2	The maximum – minimum amplitude of S1 & S2 derived from the acceleration signal.
	Differential maxima	B1 or B2	The maximum – minimum differential amplitude of S1 & S2 derived from the acceleration signal.
	Envelope	C1 or C2	Integral of the heart sound signal.
Energy	Shannon energy	D1 or D2	Attenuates high amplitude signals and provides higher weight toward low intensity content. *E* = −*x*^2^log(*x*^2^). (E=Energy||x=Signal).
	Shannon energy integral	E1 or E2	Integral of Shannon Energy.
	Shannon entropy	F1 or F2	Emphasizes medium strength amplitudes while attenuating low & high intensity amplitudes *E* = −|*x*|log|*x*|.
	Shannon entropy integral	G1 or G2	Integral of Shannon Entropy.
Temporal	S1 abs max to S2 max interval	H	Interval between maximum positive rectified S1 & S2 of the acceleration signal.
	S1 & S2 max to Vpace interval	I	The maximum amplitude location with respect to the left ventricular pacing spike was measured.
	S1 & S2 min to Vpace interval	J	The minimum amplitude (-ve peak) location with respect to the left ventricular pacing spike was measured. Interval between maximum negative rectified S1 & S2 of the acceleration signal.

The pressure analysis segmented the pressure signal during the center of the diastolic phase and extracted the largest, morphologically coherent, group of beats to account for morphology changes caused by factors such as respiration, prior systolic pressures, and independency from the ECG annotation algorithm. This allowed complete morphological assessment of the pressure curve during the cross-confirmation stage.

Following the segmentation of each heartbeat, reference features were extracted from the LV signal being LVP max and LV dP/dt max.

Only the acceleration-based features from each *Z*-axis sensor were retained because this direction is the most reproducible, being perpendicular to the epicardium, resulting in better interpretable signals, and reducing computational overhead.

The automated morphological assessment was performed on all input signals to remove irregular heartbeats. All cardiac cycles within each pacing configuration were resampled to match the most reoccurring number of samples per segment to improve the comparison of the individual beats. For each pacing configuration, the largest group of beats, providing the greatest amount of coherence were evaluated using the cross-correlation coefficient for each beat permutation (18). This comparison was performed on the ECG, pressure, and acceleration signal separately to ensure that each beat's mechanic and electronic response conform with each other. Beats that displayed large morphological deviations from the rest of the beats within each pacing setting were excluded from further processing.

### Cardiac Function Modeling

A decision tree (19–22) based machine-learning model was employed to estimate cardiac function in form of absolute LVP and/or its first derivative. The model structure consists of a multi-class classification system facilitated by decision trees. To reduce the high amount of variance demonstrated by individual decision trees, a bootstrap aggregated ensemble was used, for which several subsets of training data were used in the training of individual trees.

During the training process, acceleration feature-based rules were generated to allow optimal estimation of the absolute maximum LVP and/or its derivative. The Gini's diversity index (Equation 1) aids in maximizing information gain for each decision tree by identifying splits in the training data that reduce the probability of misclassification and hence maximize estimation performance.


(1) $$\begin{array}{r} Gini\;index\;\left(D_{p},f\right)=I\left(D_{p}\right)-\sum_{j=\;1}^{m}\frac{N_{j}}{N}I\left(D_{j}\right) \end{array}$$

*Gini index* = *identifier used to reduce misclassification probability;* *f* = *analyzed feature (subset);* *D*_*j*_ = *samples at the child node;* *D*_*p*_ = *samples of the parent node;* *I* = *current node;*

*N*_*j*_ = *total number of samples available at the current node;* *N* = *total number of samples*.

This process iteratively refined each decision tree by subdividing the training dataset into smaller sub-categories until the maximum number of splits was reached or the remaining data did not require any more subdivisions.

Feature selection consisted of an iterative process that generated multiple competing estimation models. Each of these models was based on a unique permutation of the available feature sets for their training. This result allowed investigation into the estimation potential of individual features and their potential to be complementary with secondary and/or tertiary features.

To prevent over-fitting, the number of features used in each permutation was limited to a total of three. To reduce the number of permutations, the process started with a single feature and optimized estimation accuracy or loss by selectively adding and/or replacing features until the maximum potential was reached.

Performance metrics applied in the model training process were estimation –accuracy/–loss (Equations 2 and 3). Both metrics were evaluated during 10 × k-fold cross-validation, using the mean value of all folds. The mean accuracy evaluates the number of correctly against incorrectly estimated values. Alternatively, the K-fold validation loss was used. Validation loss penalizes larger discrepancies in misclassifications significantly higher than small discrepancies. While this study primarily focused on estimation accuracy, estimation loss was used as validation to confirm model performance.


(2) $$\begin{array}{r} \text{Loss}\left(y,ŷ\right)=\frac{1}{N}\sum_{i=0}^{N}{\left(y-{ŷ}_{i}\right)}^{2} \end{array}$$

(3) $$\begin{array}{r} Validation\;accuracy=\frac{1}{N}\sum_{i=\;0}^{N}y_{p} \end{array}$$

*N* = *total number of classification attempts;* *y*_*p*_ = *correctly estimated classification;* *Loss*(*y*, ŷ) = *validation loss for single sample; N* = *total number of classification attempts;* *y* = *true classification;* ŷ = *estimated classification*.

Model validation was addressed by using the above described k-fold validation method in addition to holdout validation, to address potential issues that may arise from over/underfitting. In addition, each feature permutation used to generate an estimation model was limited to a maximum of three to prevent the chance of overfitting. Before the training of the estimation model, for the personalized model, 5% of the dataset was removed for illustrative purposes *via* holdout validation of the final model. Depending on the availability of data in each pressure category, the selection of validation samples was reduced to retain a robust training dataset. The model's performance was evaluated using the average k-fold validation whose results are shown in Table 2. The “Leave one out”/generalized method used k-fold validation to find the most performant model, while one animal was completely removed from the training set. The model was then validated with holdout estimation whose results are given in Table 3.

**Table 2:** Accuracies for estimating LVP and LV dP/dtmax at different levels of resolution and for all sensor locations.

	**Mitral valve (%)**		**LV apex (%)**		**RV apex (%)**		**Right atrium** **(%)**		**Tricuspid valve (%)**	
**Optimization method**	**Acc**	**Loss**	**Acc**	**Loss**	**Acc**	**Loss**	**Acc**	**Loss**	**Acc**	**Loss**
**Left ventricular absolute maximum pressure (range 60–130 mmHg)**										
Resolution (Interval)										
20 mmHg	96	94	93	93	93	93	94	90	94	91
10 mmHg	87	87	88	87	88	87	87	86	89	87
5 mmHg	81	78	83	79	84	80	82	75	83	82
**Left ventricular dP/dt max pressure (range 600–1,600 mmHg/s)**										
Resolution (Interval)										
200 mmHg/s	93	90	94	94	94	89	94	92	96	94
100 mmHg/s	90	90	90	88	88	88	88	88	87	86
50 mmHg/s	86	85	86	85	85	84	86	85	86	84

*Optimization methods: (Acc): accuracy optimization || (Loss): loss optimization. All values are evaluated using average 10 × k-fold validation results from the respective estimation models. LV, left ventricular; RV, right ventricular*.

**Table 3:** Accuracy optimization using holdout validation results generated by the “Leave one out” method.

**LV dPdt(max)** **200 mmHg/s**	**Mitral valve (%)**	**Left ventricular apex (%)**	**Right ventricular apex (%)**	**Right atrium (%)**	**Tricuspid valve (%)**
Leave out Animal 1	83	81	80	81	82
Leave out Animal 2	90	85	81	83	87
Leave out Animal 3	82	79	82	80	82
Leave out Animal 4	71	69	67	73	74
Leave out Animal 5	81	84	78	79	77

*Each row indicates a model that excludes and was validated on the animal listed in the first column*.

During the modeling process, each feature permutation underwent k-fold validation which ensured that rules generated on subsets of data were applicable to the remaining dataset.

### Statistics

We have performed a one-way analysis of variance (ANOVA) for testing significance (p < 0.05) between bin-size (low-medium-high) as well as between personalized and generalized models in both accuracy and loss estimations.

## Results

**Figure 4:**
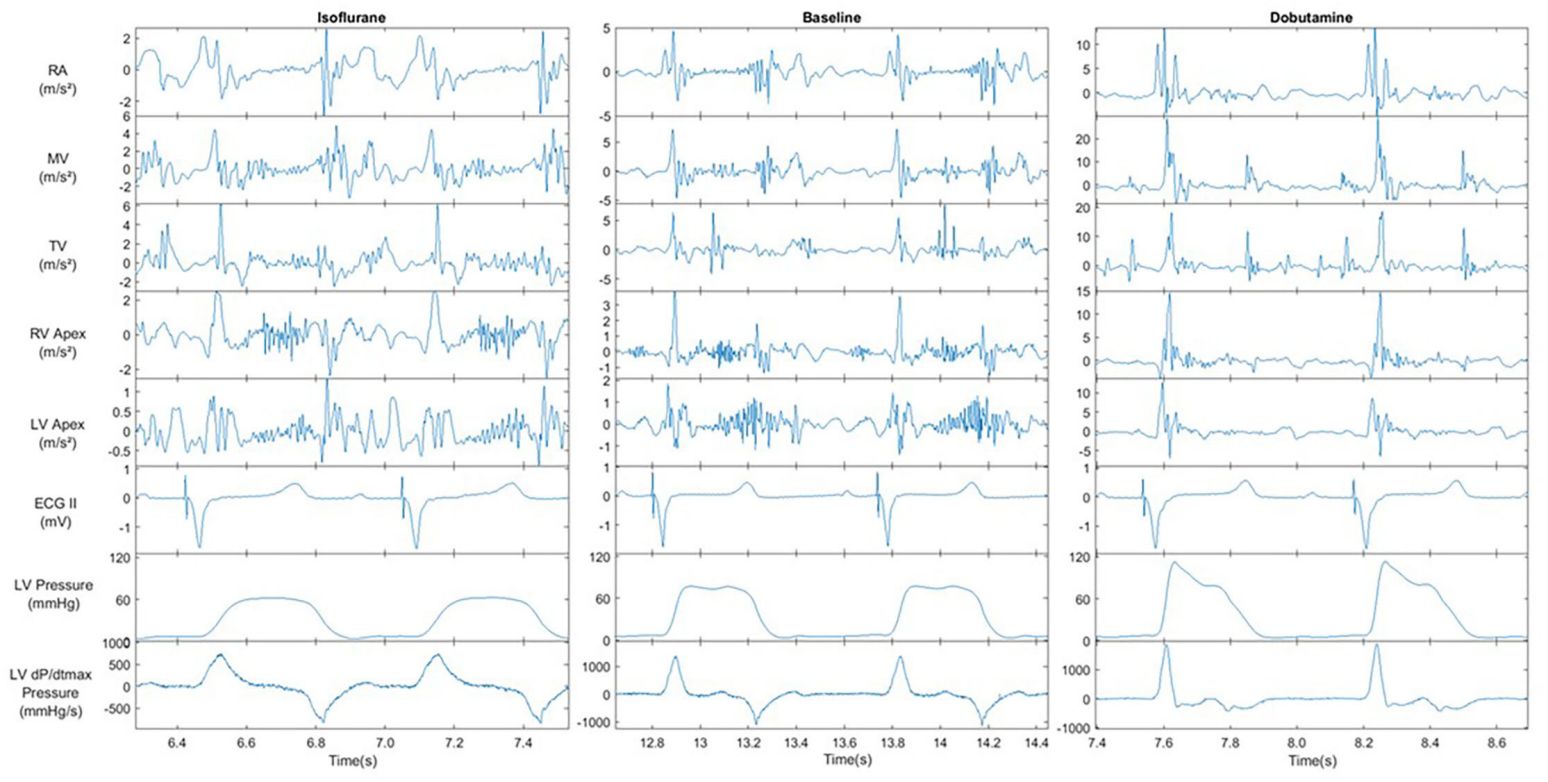
Recorded signals of Left ventricular pressure, ECG and all acceleration recording sites under the influence of cardiovascular modifiers. All signals were recorded @BIV | AVd = 150 ms | VVd = 0. Acceleration signal show the result of the X, Y and Z axis magnitude filtered between 1-150 Hz. AVd = atrio-ventricular delay, VV = intra-ventricular delay.

Figure 4 displays an example of the acquired signals during the 3 hemodynamic steady states: baseline, isoflurane, and dobutamine. The profound negative and positive effects on hemodynamics can be seen in isoflurane and dobutamine, respectively. Also, the specific acceleration signals of the 5 anatomic sensors and their response to changing hemodynamics are illustrated in Figure 4.

Post-experiment segmentation processing resulted in a dataset of over 6,000 cardiac cycles that consisted of a complete annotated set of training and reference data. The final selection of the best-performing models is listed in Table 4. Of all initially proposed features in Table 1, acceleration amplitude and/or energy-based features proved to correlate best to the hemodynamic variables; with S1/S2 maximum amplitude and S1/S2 integral appearing most frequently in the final models. In contrast, time-based features were widely neglected and only the feature expressing the duration between the maximum amplitude of S1 and S2 acceleration signals showed any re-occurrence in high-performing models.

**Table 4:** Most relevant features according to their re-occurrence when generating accuracy optimized models.

	**LV Pressure (max)**			**LV dP/dt (max)**		
**Sensing location**	**Feature 1**	**Feature 2**	**Feature 3**	**Feature 1**	**Feature 2**	**Feature 3**
Mitral valve	C2	B1	C1	G1	C1	C2
Left ventricular apex	A1	C1	C2	C2	A1	C1
Right ventricular apex	E2	A1	C1	A1	G1	G2
Right atrium	C1	G2	A1	G2	H	C1
Tricuspid valve	C2	C1	A1	A1	C1	C2

*Feature explanations are given in Table 1. 1 and 2 designations refer to the acceleration-based equivalent to the occurrences to S1 and S2 heart sounds*.

**Figure 5:**
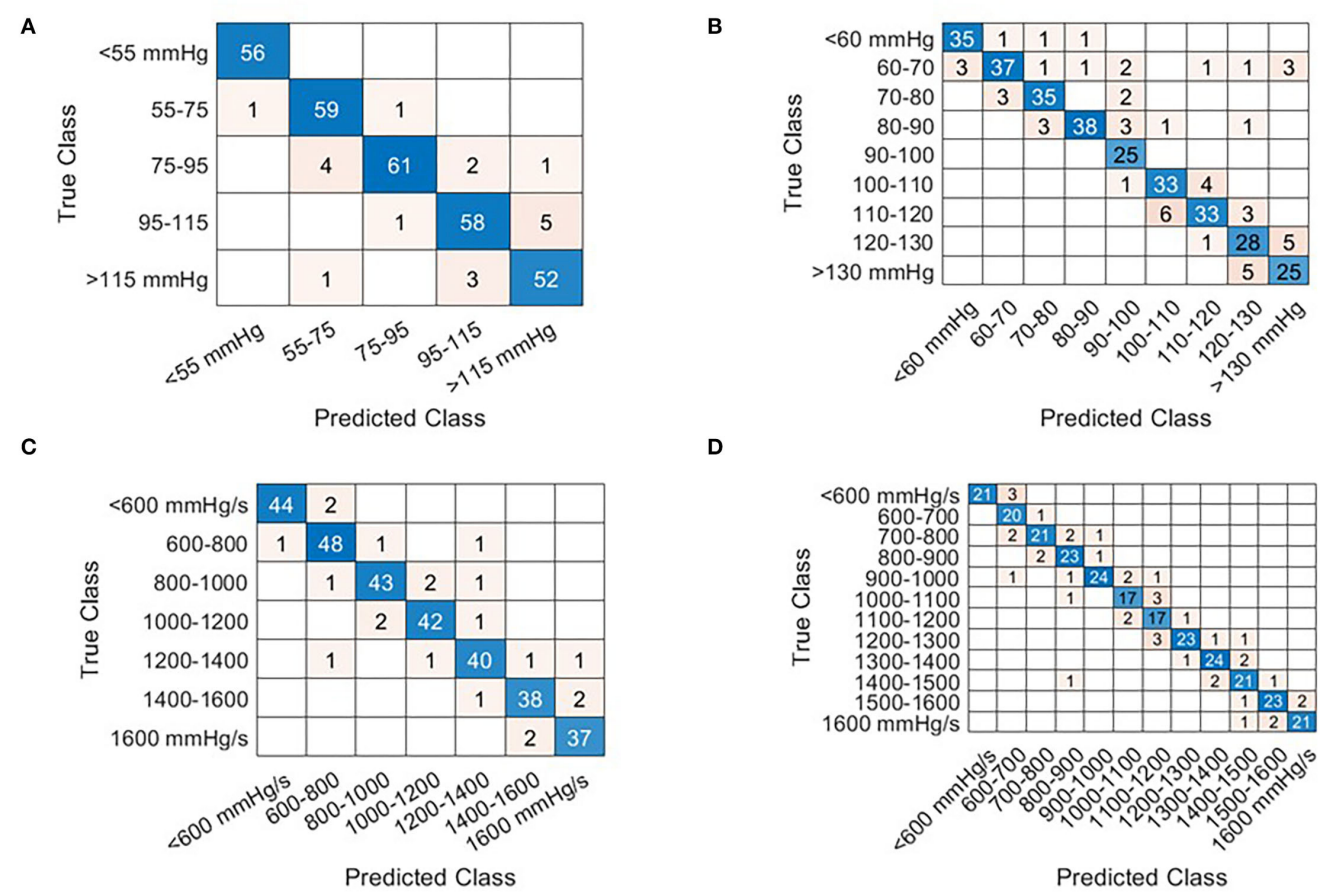
Confusion matrices that illustrate the correctly and incorrectly estimation results for each given beat via holdout validation from the Mitral valve sensor. The row of the matrix corresponds to the true class while columns correspond to the predicted class. Diagonal entries correspond to correct estimates while off-diagonal entries represent incorrect estimates. The beats selected for holdout validation were selected at random. At low prevalence of cardiac cycles in any given category, the number of selected samples is reduced in favor of the training dataset. Examples are shown for LVPmax [**(A,B)** Interval bins of 20 and 10 mmHg respectively] and LV dP/dt(max) **(C,D)** interval bins of 200 mmHg/s, **(D)** 100 mmHg/s.

Examples of the model performances are depicted in Figure 5 which shows the result of four models tested under holdout validation with data extracted from the sensor in the accelerometer in the proximity of the mitral valve. Each row represents the holdout validation results of two models, with increasing resolution, estimating LVPmax and LV dP/dt max, respectively.

Table 2 displays the results obtained for all sensor locations for both estimation accuracy and loss optimization and three levels of resolution for LVP and LV dP/dtmax. Accuracies for LVP with bin-size of 20 mmHg ranged between 90 and 96%, for bin-size of 10 mmHg between 86 and 89%, and for bin-size of 5 mmHg between 75 and 83%, with small non-significant differences between the sensor locations. Similar results were obtained for LV dP/dtmax using differences of 200, 100, and 50 mmHg/s, wherein accuracy ranges of 89–96%, 86–90%, and 84–86%, were achieved respectively. An overview of the results is given in Table 2.

Bin-size significantly (*p* < 0.001) affected both accuracy and loss estimations, indicating increased accuracy/loss at larger bin-size.

An additional investigation was performed using the “Leave one out” method, which validates estimation models, that are trained on N-1 participating subjects, against the remaining subject. Using the lowest estimation resolution for the estimation of LV dP/dtmax, an average estimation accuracy evaluated across all sensing locations was 80% with an SD of 5.4% (Table 3). “As expected, there was a significantly (P < 0.01) lower estimation accuracy in the “leave-one-out”/generalized model than the personalized model, comparing both at a bin-size of 200 mmHg/s.”

The holdout validation figures (in Table 3) indicated about a 15-percentage point lower accuracy than the k-fold validation results listed in Table 2.

## Discussion

This study provides the proof of principle for a novel method for estimating absolute LVPmax (and LV contractility) using machine learning analysis of epicardial accelerometer signals. The majority of features that contributed to our prediction models were related to the amplitude and energy of the accelerometer signal and very few related to the timing of them. Accuracies were similar for all five sensor locations.

### Comparison to Other Studies

These results significantly extend the application of mechano-sensors in estimating cardiac function, which is so far largely limited to optimization of pacemaker settings by the SonR system (15, 23), without the knowledge of absolute values of pressures. Another study on pigs showed that an epicardially placed accelerometer can be used to assess changes in preload, and so filling status, using the frequency of myocardial acceleration (24). In preclinical studies, Thakur et al. showed that analysis of S1 and S3 amplitude signals from accelerometers integrated into an implanted pacemaker device was able to monitor the change of absolute left atrial pressure over time (25). While these studies showed the relation between accelerometer signals and hemodynamic status, this study, to the best of our knowledge, is the first to show that absolute LV pressure and contractility levels can be derived from accelerometers. Moreover, the finding that such estimations can be obtained from various (atrial, right, and left ventricular) locations is novel. Potentially, the introduction of additional features derived from, e.g., frequency components or gyroscopic signals (easily imbedded in current accelerometer sensors) may contribute positively to our developed pressure estimation model (25, 26).

### Possible Need for Personalization

Two ways of model development were used, namely, a personalized model (including all available subjects' data) and a generalized model (leave-one subject's data out). In the former approach, k-fold validation from all 5 individuals was used while holdout validation was used for the latter. The approach clearly shows the proof of concept. However, when applying this approach to the clinical situation it would require a period of validation/calibration with a gold-standard measurement before continuing with the mechano-sensor information only (see below). Because such an approach may not always be possible or desirable, the second option shows that using the information of, in our case four individuals, the mechano-sensor information of the fifth individual is still quite reliable. In the clinical situation, such a development-set is likely to be considerably larger than four, which can be expected to significantly increase the accuracy of the leave-out approach in the clinical setting. With either approach, it may also be required to create different prediction models for the various sensor locations. While accuracy was comparably high for all sensor locations, the optimal three features to reach this result differed to some extent. This “location independence” is reflected in the results wherein differences in recording locations showed only minute changes in estimation performance while selecting dissimilar features (see Table 4).

### Further Possible Applications/Integration

The proposed method may be used in general hemodynamic monitoring applications at low-to-moderate resolution (e.g., intensive care unit). Secondly, it may be used to track cardiac function to detect decompensation in an early stage. The currently developed method allows for simple integration into embedded software of monitoring and other (e.g., pacemaker, ICD) devices (27). With respect to the latter, our method may assist in identifying life-threatening arrhythmias by adding the hemodynamic analysis on top of the current electrophysiological analysis in these devices to prevent unnecessary defibrillatory interventions (28, 29).

While the present study used epicardial sensors, the approach used in this study may also be applied to less invasive acceleration measurements, such as heart sounds measured on the skin, or microphones or accelerometers mounted on or inside implanted devices or pacing/defibrillation leads. Calibration steps can be either procedure-related or added to the procedure and can be either invasive (like in the current study) or non-invasive, e.g., using a common pressure cuff or finger plethysmography as shown for CRT patients (30, 31). Different calibration circumstances can also be envisioned such as rest/exercise/pacing intervention in case of an implantable pulse generator placement used in CRT. Supplementary data of patients, with similar conditions and equipment setup, can be used to improve the robustness of the model during implantation or any follow-up session.

The processing pipeline, used for an automated beat and feature selection, may be of clinical use. The implemented method for synchronization of acceleration signals and ECG accounts for differences in sampling rates and standardizes the acquisition process before analysis. The prerequisite for this integration was limited to a single auxiliary recording channel with a synchronization pulse. This pipeline may also be useful for the rapid generation of estimation models with new data, including those in patients during a period of monitoring or the implantation of pacemakers or other monitoring devices. Scalability of estimation models allows accommodation of device constraints such as processing overhead, battery life, and cost. A tradeoff exists between model complexity and its estimation accuracy, wherein a reduction in complexity may cause a reduction in estimation accuracy and vice versa [i.e., the use of neural networks (17, 32)].

For specific clinical applications, the method of feature acquisition post-implantation may have to be altered in case ECG, which is used as the reference signal, is not available. Alternative sources of reference can be used post-implantation as long as they include timing information about the cardiac cycle. Preferred alternatives would be the use of “surrogate ECG” provided by a defibrillator, EGM feedback, or timed pacing intervals.

### Limitations of the Study

Some limitations of the study should be noted. First of all, the acquisition of mechanosensory data was performed under preclinical conditions in normal porcine hearts with an open chest. Clinical application in patients with compromised heart function should be considered with caution because several differences may apply, like differences in cardiac contractile force, the influence of the open- or closed-chest as well as differences in sources of noise. These noise sources were kept to a minimum in the controlled experimental environment, whereas in the real-life situation, environmental noise and movement artifacts may be higher because in patients with heart failure also lung sounds may become stronger. In addition, while an extensive experimental protocol with a generous variety of interventions was performed, only five animals of similar weight and dimensions were used in this trial. This may reduce the signal variability in comparison to the amount experienced in a clinical setting. Finally, the personalized model may learn to identify the animal instead of the pressure-derived estimates, therefore risk mitigation methods such as k-fold validation and ensembling are employed. In addition, the numerous (pacing) configurations provided a considerable range in LV max pressure and LV dP/dt max values. To further reduce this known phenomenon of machine learning, it is recommended that future investigations increase the number and physiological conditions of the subjects in the training set.

To adhere to the processing limitations of (cardiac) pacing devices, only features requiring little computational overhead were used. This limited feature selection to the temporal domain wherein only amplitude, timing, and energy-based features was considered. Expansion of potential features with more complex acquisition processes may further improve results.

## Conclusion

The use of epicardial accelerometer data in combination with a bootstrapped decision tree ensemble algorithm can reliably estimate absolute hemodynamic statuses, such as intracardiac left ventricular pressure and its derivative in a controlled preclinical setting. The algorithm is simple enough to be scaled with low computational requirements to be used for monitoring cardiac function by a simple computer, microcontroller, or dedicated integrated circuitry.

## Data Availability Statement

The datasets presented in this article are not readily available because data are owned by Maastricht University and Medronic, plc. Requests to access the datasets should be directed to richard.cornelussen@medtronic.com.

## Ethics Statement

The protocol was approved by the Central Committee for Animal experiments (CCD) in the Netherlands and the Animal Experimental Committee of Maastricht University.

## Author Contributions

PW, HL, MK, FP, and RC designed the research studies. PW, MK, LH, and HL carried out the studies. PW and LH analyzed the data. PW wrote the paper with help from HL, TD, FP, and RC. RC had primary responsibility for the final content. All authors read and approved the final manuscript.

## Funding

This project has received funding from the European Union's Horizon 2020 Research and innovation program under the Marie Sklodowska-Curie Grant Agreement: No 764738 (Personalized *In-silico* Cardiology (PIC).

## Conflict of Interest

PW and RC are Medtronic employees. The remaining authors declare that the research was conducted in the absence of any commercial or financial relationships that could be construed as a potential conflict of interest. The reviewer ER declared a past co-authorship with the RC to the handling editor.

## Publisher's Note

All claims expressed in this article are solely those of the authors and do not necessarily represent those of their affiliated organizations, or those of the publisher, the editors and the reviewers. Any product that may be evaluated in this article, or claim that may be made by its manufacturer, is not guaranteed or endorsed by the publisher.

## Abbreviations

AV: atrio-ventricular
BiV: biventricular
DOB: dobutamine
ECG: electrocardiogram
ISO: isoflurane
LV: left ventricle
LVP (max): left ventricular (maximal) pressure
LV dP/dtmax: maximum rate of rise of LVP
VV: intraventricular.
