# Supplementary material for: Left Ventricular Pressure Estimation Using Machine Learning-Based Heart Sound Classification
Source: Front Cardiovasc Med. 2022 May 25;9:763048. doi: 10.3389/fcvm.2022.763048 (PMC9174571; doi:10.3389/fcvm.2022.763048)
Supplement: Supplementary file 1 [file Data_Sheet_1.docx]

**Supplement 1**

**Signal analysis/Pre-processing**

**Overview**

Machine learning based algorithms require large quantities of samples in order to train and validate the estimation models. For this purpose, a beat-to-beat analysis method was implemented to autonomously process the available electrocardiogram, pressures, and acceleration data with the purpose of creating a training dataset for machine learning.

To minimize the interdependence of signals in the analysis Pressure and ECG are processed independently from each other. Once all signals were segmented into individual cardiac cycles, the results were merged to identify discrepancies such as artifacts, double or missed beats and therefore verify the quality of each individual beat. Beats that did not qualify (based on criteria discussed in a later section) in Acceleration, ECG and Pressure morphology were removed from the dataset automatically.

**Cardiac cycle validation**

The process for estimating signal quality was performed for all signals and occurs after segmentation of the signal into cardiac cycles. Pacing configurations are processed individually to reduce the variability of the signal. For each configuration the largest coherent group of cardiac cycles are indexed for further processing.

Using this method, we made the following assumptions:

1. The signal was not completely random.
2. The largest coherent group of cardiac cycles within the recording was the correct signal that was meant to be segmented and annotated.
3. The signature occurs at least twice, provided that more than a single repeated instance of the signature was included in the signal.

Using the previously evaluated ECG analysis, the detected timing of atrial pacing was used to segment each beat. To improve comparison results of the annotated beats by standardization, each beat was resampled to match the sample size of the beat with the greatest number of samples.

Each beat was split into multiple overlapping segments which are grouped based on their segment order for all the beats. The cross-correlation coefficient is evaluated for each group giving an indication of similarity between the beats within each segment group. Taking the median of the cross-correlation coefficients (as evaluated with the formula below; Formula (E1) of all the groups for each beat allows identification of most similar/dissimilar morphology within any given pacing setting and therefore enables extraction of the largest coherent group of cardiac cycles to be identified.

$C_{xy}(m)=E\{x\left( n+m \right)y^{'}\left( n \right)\}$ **Equation 1**

$\hat{C}_{xy,coeff(m)}=\frac{C_{xy}}{\sqrt{\sum\left| x \right|^{2}*\sum\left| y \right|^{2}}}$

**E1**: $C_{xy}$ *= True correlation result of two signals; x = Signal 1; y = Signal 2;* $y^{'}$ *= complex conjugation of signal;* $\hat{C}_{xy,coeff(m)}$ *= correlation coefficient result of tw o signals; E = mathematical expectation.*

Beats identified to show too much deviation from the beat representative, are removed along with its affiliated pressure and ECG values.

**Electrocardiogram Analysis**

The electrocardiogram analysis process aims to fulfill two tasks, the first of which was the validation of signal quality used to select cardiac cycles for feature extraction. The second task was the annotation of relevant morphology landmarks such as atrial and ventricular pace for the extraction of time dependent training features and to facilitate segmentation of the acceleration signal.

Before ECG was segmented and training features are extracted pacing spikes are removed from the signal. This was performed by annotating the pacing spikes with the help of a 200Hz high pass filter and a peak detector. In case multiple ventricular pacing spikes are detected, caused by an intraventricular delay during bi-ventricular pacing, only the leading spike was considered to be responsible for inducing the majority of tissue depolarization.

Pacing spikes are detected using a 200Hz high pass Butterworth filter with four poles to accentuate the high frequency components such as pacing spikes while removing lower frequency components. The filtered signal is used to evaluate its Shannon energy with the formular illustrated below in equation below (See table 1), which is the average spectrum of the signal’s energy. This results in oscillations of medium to high amplitude to become more pronounced as low amplitude portions of the signal are attenuated.

Sensing of right atrial pacing, during the experiment, allows identification of atrial pacing spikes, leaving only Ventricular pacing spikes to be annotated. It is noteworthy that only a single ventricular pacing spike is annotated for each beat.

In case multiple ventricular pacing spikes are detected, caused by an intraventricular delay during bi-ventricular pacing, only the leading spike is considered to be responsible for inducing the majority of tissue depolarization. Thresholding was used to determine the onset and offset of each pacing spike. The threshold was based on 5% of the maximum (non-outlier & specific to V/A-Pace) amplitude. Each positive and negative transition of the signal passing the threshold was attributed to the onset and offset of the pacing spike respectively.

A 3-45Hz second order Butterworth bandpass filter was applied to the raw signal to remove frequency noise and drift caused by respiration. Previously evaluated onsets & offsets were selected with a 10ms window. The trajectory of the selected samples was used to estimate the intermediate signal via interpolation. The interpolated section was subsequently used to replace detected pace spike. Windowing around the ventricular pacing spike, a R-peak was detected.

Finally, the ECG signal was segmented into individual cardiac cycles by from A-A-Pace and the aforementioned Cardiac cycle validation was applied to select the largest coherent group for further processing.

**Pressure analysis**

Similarly, to the ECG analysis, the pressure analysis process was used to identify valid beats for the machine learning process.

A 1Hz high pass filter was applied to the raw signal, remove to drift as well as centering the signal around zero. Occurrences of the filtered signal transitioning a zero threshold were detected using simple comparison operators. Approximately half the distance between the transition points are indexed to be used as start and stop in the cardiac cycle validation.

In addition, to the signal quality check, pressure features are extracted as classification features for the machine learning models. The quality check was applied here to only retain beats of consistent quality and to remove “double” or missed beats from further processing.

*Accelerometer analysis*

Acceleration uses atrial-pacing spikes to segment the signal into cardiac cycles prior to Cardiac cycle validation, after which the results of the signals are merged in the cross-evaluation process.

*Cross evaluation*

Cross evaluation was performed between the annotated ECG, pressure and acceleration signal. Indexed beats are assigned features from both the ECG analysis as well as the pressure analysis process. Beats identified to be incomplete or mismatched, are removed from the dataset.

*Electrocardiogram Feature Extraction*

Electrocardiogram analysis was performed following the validation processes, wherein each beat was analyzed for time-based classification features that will be used as training features in the training process, which are already elaborated upon in Table 1 (in the original manuscript).

*Pressure Feature Extraction*

Features (LVPmax and dP/dtmax) annotated in the pressure signal are used as reference in the training process.

*Accelerometer Feature Extraction*

With the aid of previously evaluated timings of LVdP/dtmax and LVdP/dtmin, two windows are segmented in the accelerometer signal around A1 and A2, respectively. A1 segments range from R-Peak to LVdP/dtmax+130ms while A2 segments are entirely based on A2 which range from LVdP/dtmin-50ms up to LVdP/dtmin +100ms

*Model overview*

After completion of the feature extraction of ECG, pressure and acceleration signals, a machine learning algorithm was used to analyze the data. Estimation of LVPmax and LV dP/dtmax was performed by a bagged decision tree model. Bagged tree models, also called Bootstrap-aggregated models, are ensemble methods which utilize multiple decision trees in order to make a decision/prediction.

Decision trees are branching hierarchical structures consisting of nodes representing chained conditionals. Bootstrap aggregation aims to reduce the variance of single decision trees, for which several subsets of training data are used to create a collection/ensemble of decision trees [1].

| _1_ | Loh, W. (2002). Regression Trees With Unbiased Variable Selection and Interaction Detection. *Statistica Sinica* 12**,** 361-386. |
| --- | --- |

The completed ensemble forms a consensus during which the result is averaged to increase the prediction accuracy. This results in a more complex decision-making process compared to simple decision trees; however, the bootstrap-aggregated model becomes more robust in its decision-making process by improving stability, reducing variance and increasing accuracy as illustrated in the equation (E2) below.

$f bag(x) = \frac{1}{B}\sum_{b=1}^{B} f^{b}(x)$ **Equation E2**

***E2:*** *f bag(x) = Averaged ensemble decision based upon the decision of individual trees (*$f^{b}$*).
B = number of trees accommodated by the ensemble.*

Each of these trees contained in the ensemble, are grown independently from each other in form of a weak learner. The number of learners was set to a static 30 to keep complexity controllable and to make the models more comparable to each other. The weak learners used in this model attempt to generate a set of rules whose accuracy, in estimating pressure, is above random chance.

Differences in rulesets between the weak learners are due to the random selection of observations, used in the training process. As part of Bootstrap aggregation, observations contained in the training data, may be used for one or more learners in order to generate rules. During classification, the bootstrapped ensembles form a consensus during which the individual trees make individual decisions that is used to evaluate the majority vote.

**Model Training**
To train the models to use acceleration features in its estimation process, the be estimated pressure features require to be discretized. The discretization process involves categorization of the pressure data based on their individual amplitudes, during which beats of similar pressures are grouped into the same category. Due to the previously performed beat to beat analysis, each annotated pressure value (LVPmax and dP/dtmax) is accompanied by a complete set of acceleration features (listed in table 1) which will be used as reference to generate rules capable of estimating pressure.

*Learning process*

***Feature selection***
The experiments employed multiple sensors in proximity to different cardiac structures. Due to the variation in both location and orientation to the vibration sources, features extracted from each sensor may provide dissimilar predictive potentials. Thereto, feature selection was automated. However, given the large number of potential training features available for prediction, creating models based on every possible permutation of features would be too computationally heavy.

Feature selection has been implemented in the form of an iterative process, that attempts to improve upon previously generated models by altering its training features. During the initial iteration, the selection algorithm generates individual models by using each available feature separately as training set. This results in a single bagged tree ensemble for each available feature. The model with the greatest predictive potential is evaluated and its training feature is included in the training set of subsequent iterations.
After each iteration, the algorithm is given three actions that it can execute. The first two actions aim to improve upon the previous models while the third action terminates the iteration process.

1. **Replacement** of features is the first action the algorithm will take in order to improve the model. Provided that at least two features are included in its current training set, permutations of the current training set are performed with the rest of the available feature pool. Only a single feature contained in the existing training set may be replaced per permutation.
2. **Growth** of the current number of features in the training set will be performed if replacement of features has not been successful in improving the model. If the maximum number of features has not yet been reached and replacement of features has not improved the model, an additional feature is added to the already selected training features.
3. **Termination** of the feature selection algorithm is induced in case the model has reached its peak performance or a failure condition has been recognized. Reasons for termination include:
   - Reaching its maximum potential as evaluated by prediction accuracy and/or classification error wherein the newly generated models show no improvement in its capabilities over the previous model. This does not prerequisite reaching a specific number of features and may even terminate after selecting only a single feature.
   - Reaching the maximum number of features that it is allowed to include in the training features.
   - The algorithm has no more features available in the feature pool to choose from.

*Over and Underfitting*

Modelling methods, used to make predictive decisions/estimations, can negatively affected by over/under-fitting. Although overfitting is most prevalent in machine learning, both overfitting and underfitting can contribute to poor performance in machine learning applications. To prevent over and under-fitting we used hold-out-validation as well as K-fold cross validation.

Additionally, the iterative process was set up to limit its feature selection to a maximum of three.
